# Understanding Engagement and the Potential Impact of an Electronic Drug Repository: Multi-Methods Study

**DOI:** 10.2196/27158

**Published:** 2022-03-30

**Authors:** Charlene Soobiah, Michelle Phung, Mina Tadrous, Trevor Jamieson, R Sacha Bhatia, Laura Desveaux

**Affiliations:** 1 Institute for Health System Solutions and Virtual Care Women's College Hospital Toronto, ON Canada; 2 Institute for Health Policy, Management & Evaluation University of Toronto Toronto, ON Canada; 3 Leslie Dan Faculty of Pharmacy University of Toronto Toronto, ON Canada; 4 Unity Health Toronto Toronto, ON Canada

**Keywords:** centralized drug repository, mixed methods, electronic survey

## Abstract

**Background:**

Centralized drug repositories can reduce adverse events and inappropriate prescriptions by enabling access to dispensed medication data at the point of care; however, how they achieve this goal is largely unknown.

**Objective:**

This study aims to understand the perceived clinical value; the barriers to and enablers of adoption; and the clinician groups for which a provincial, centralized drug repository may provide the most benefit.

**Methods:**

A mixed methods approach, including a web-based survey and semistructured interviews, was used. Participants were clinicians (eg, nurses, physicians, and pharmacists) in Ontario who were eligible to use the digital health drug repository (DHDR), irrespective of actual use. Survey data were ranked on a 7-point adjectival scale and analyzed using descriptive statistics, and interviews were analyzed using qualitative descriptions.

**Results:**

Of the 161 survey respondents, only 40 (24.8%) actively used the DHDR. Perceptions of the utility of the DHDR were neutral (mean scores ranged from 4.11 to 4.76). Of the 75.2% (121/161) who did not use the DHDR, 97.5% (118/121) rated access to medication information (eg, dose, strength, and frequency) as important. Reasons for not using the DHDR included the cumbersome access process and the perception that available data were incomplete or inaccurate. Of the 33 interviews completed, 26 (79%) were active DHDR users. The DHDR was a satisfactory source of secondary information; however, the absence of medication instructions and prescribed medications (which were not dispensed) limited its ability to provide a comprehensive profile to meaningfully support clinical decision-making.

**Conclusions:**

Digital drug repositories must be adjusted to align with the clinician’s needs to provide value. Ensuring integration with point-of-care systems, comprehensive clinical data, and streamlined onboarding processes would optimize clinically meaningful use. The electronic provision of accessible drug information to providers across health care settings has the potential to improve efficiency and reduce medication errors.

## Introduction

### Background

Pharmacological management of chronic diseases and acute illness is common, with over 41% of Canadians reporting using at least one prescription drug [1]. The lack of comprehensive medication information at the point of care can increase the likelihood of an adverse drug event (ADE) [2,3]. A meta-analysis of 46,000 patient records estimated that 20% of hospital inpatients in high income countries have at least one ADE, of which 32% are deemed preventable [4]. In community settings, 5% of patients experience at least one ADE in their lifetime [5], which translates into 16 million Americans or 2 million Canadians based on current population estimates [6,7]. ADEs are often caused by preventable prescribing errors (ie, wrong dose or therapeutic choice) and ineffective monitoring of pharmaceutical care [5,8], costing health care systems an estimated CAD $1.1 billion per year (US $0.89 billion) [9].

Medication reconciliation reduces the likelihood of ADEs [10] and involves a collaborative effort between clinicians and patients to establish a best possible medication history (BPMH) to improve medication safety [11,12]. Medication histories reported by patients during the BPMH process are often verified using secondary sources of information, such as pharmacy or discharge records [13]; however, this process is time consuming and cumbersome at the point of care.

There are several examples of national and international drug repositories, such as the British Columbia Pharmanet [14] or Sweden’s National Prescription Drug record [15], which typically contain dispensed medication information, such as the drug name, the dose, allergy, and intolerance information, which can be valuable when constructing a BPMH. A few drug repositories also include information on prescribed medications and private insurance claims to facilitate decision-making at the point of care [16,17]. Access to a centralized drug repository can support the BPMH process and, in turn, reduce inappropriate prescribing [18], improve medication adherence [19], and reduce health care costs [20,21].

### Objectives

To achieve these aims, the Ontario Ministry of Health implemented the digital health drug repository (DHDR). The DHDR contains information on publicly funded drugs in Ontario, pharmacy services, and dispensed monitored drugs (ie, narcotics and controlled substances). The DHDR was developed by a provincial agency responsible for creating a public electronic health record system (eHealth Ontario), and access is enabled through one of two provincial clinical viewers (*ClinicalConnect* and *Connecting Ontario*), which provides access to a range of health system data assets. The DHDR was implemented in 2016 and had over 150,000 registered users across over 546 sites at the start of this study (November 2018). Eligible users of provincial viewers include clinicians who require patient medical information as part of the provision of care (eg, physicians, nurses, and pharmacists). Use statistics estimate that approximately 3000 users access the DHDR daily; however, the drivers of engagement and whether the DHDR is achieving its objectives remain unclear.

The overarching aim of this work is to examine the use, drivers of engagement, perceived value, and potential impact of the DHDR. The specific objectives are to (1) understand the perceived clinical value of accessing medication history via the DHDR, (2) identify enablers of and barriers to adoption among eligible users, and (3) understand for which clinician groups the DHDR has current or future potential clinical value and how that value is (or might be) realized.

## Methods

### Overview

A multi-method approach was used to elicit feedback from users and nonusers of the DHDR and included a cross-sectional electronic survey and semistructured interviews. The approach allowed us to simultaneously collect a breadth of responses from users and nonusers in the cross-sectional survey, whereas the interviews explored more fulsomely individual experiences with the DHDR. Interviews and the survey were conducted concurrently to maximize research efforts, and the results were triangulated and interpreted simultaneously (ie, the qualitative findings were used to help understand the survey findings and vice versa). Informed consent was obtained electronically for participants completing the survey, and verbal consent was obtained for those participating in interviews.

### Ethics Approval

Research ethics approval was obtained from the Women’s College Hospital using the assessment process for quality improvement projects (WCH APQIP REB #2019-0038).

### Study Setting

In Ontario, some prescription drugs were publicly covered by the Drugs and Devices Division of the Ministry of Health (formerly Ontario Public Drug Programs). These include the Ontario Drug Benefit Program for residents aged >65 years, the Trillium Drug Program for residents with high medication costs in relation to household income, and other specialized drug programs for those with complex conditions, such as cancer, inherited metabolic disorders, and those receiving home care [22]. In 2013, over 2.8 million, or approximately 20% of residents, received benefits through these programs to reduce the cost of medications [22]. In addition, the DHDR incorporates the provincial Narcotics Monitoring System, which captures dispensed narcotics (ie, opioids) and controlled substances (ie, methylphenidates, benzodiazepines, and barbiturates) for all residents [23]. The DHDR includes dispensed medication information from the Drugs and Devices Division Programs and the Narcotics Monitoring System; however, it does not include medications paid through private insurance companies; over-the-counter medications; or general purchase medications (eg, acetaminophen), supplements, or medication samples (ie, novel anticoagulants) provided by pharmaceutical companies.

To centralize access to provincial digital assets, the DHDR was embedded into 2 pre-existing clinical viewing portals. These portals allow providers access to a range of patient-level information, including diagnostic imaging reports, dispensed medications, laboratory results, hospital visits, and home and community care information (ie, referral details, risk assessments, and care plans) in Ontario. Although participants’ perceptions of the DHDR were influenced by their experience with the clinical viewer, exploring its functionality was beyond the scope of this study.

### Participant Recruitment

Clinicians from all sectors of the health care system were eligible for participation in the survey or interviews, provided they were clinicians who required access to electronic patient records as part of the provision of clinical care. This group includes physicians, specialists, nurses, pharmacists, and other allied health providers employed at health organizations. Allied health professionals employed in the private sector such as physiotherapists, occupational therapists, and paramedics were not eligible.

Recruitment for the survey and interviews was conducted concurrently using a multipronged approach, as no mechanism existed to identify or contact current users directly. Our first strategy involved recruiting participants through Local Registration Authorities (LRAs) working with eHealth Ontario. LRAs are individuals nominated by their organization or site to train users on clinical viewers (ie, *Connecting Ontario* and *ClinicalConnect*) and its connected repositories such as the DHDR. Using aggregate use data obtained from eHealth Ontario, we stratified the data based on the type of health care setting (ie, acute care, long-term care, and community care) and region (eg, Southwest, Northeast, and Central Ontario). Our goal was to recruit from sites with a larger pool of users to maximize the potential for recruitment. Thus, sites with fewer than 20 registered users were excluded (n=308). LRAs were offered a modest honorarium to acknowledge their efforts, and we were limited to include a maximum of 24 sites based on our funding. These sites were selected using a random number generator of the included sites with >20 registered users (n=112). The LRAs from the 24 targeted sites were then asked to distribute recruitment emails.

As the survey was constructed for the purpose of this study, there was no sample size calculation behind our target recruitment. Overall, the selected target sites had approximately 6794 active users and over 15,000 authorized users. We anticipated the LRAs would send the link to the survey to at least 50% (3397/6794) of active users, of which we estimate 30% (1019/3397) would open the email and 20% (203/1019) of which will complete the survey [24,25].

To increase participation, our secondary strategy involved recruitment through the DHDR Clinical Working Group, a group of clinicians who are actively engaged in digital health solutions at their respective organizations and advise eHealth Ontario on how to improve the DHDR. The DHDR working group members circulated a recruitment email detailing the project, contact information of our study team, and a link to the electronic survey through their internal networks. Finally, we recruited individuals through our internal networks and social media outlets (Twitter and LinkedIn). Participants who were interested would click the link to the survey or contact the study coordinator to participate in an interview.

### Data Collection

#### Cross-sectional Survey

A web-based cross-sectional survey targeted authorized users and nonauthorized potential users of the DHDR to understand their knowledge related to the DHDR and how they use its data where applicable. Nonauthorized users are individuals who are eligible for DHDR access but have not been issued secure credentials for access to the repository (ie, still not registered for access through eHealth Ontario via a clinical viewer).

Survey items were informed by previous Ministry of Health benefits and evaluation reports and past surveys conducted by eHealth Ontario. The questions were further modified and reviewed by stakeholders at the Ministry of Health and eHealth Ontario to ensure sufficient alignment to inform decision-making. In addition, to ensure we included questions relevant to our evaluation, face validity in relation to our study objective was assessed using the Clinical Sensibility Questionnaire (Multimedia Appendix 1) [26]. Three stakeholders from eHealth Ontario, 4 clinicians, and 1 researcher (outside of the research team) from Women’s College Hospital reviewed the items for inclusion. A total of 10 questions were removed to eliminate redundancy, and several questions were rephrased for clarity based on feedback.

Survey items included a demographic questionnaire followed by questions on the perceptions of the DHDR (Multimedia Appendix 2). DHDR users rated their experience across four key domains: (1) usefulness, (2) quality of data, (3) training, and (4) satisfaction. Participants also rated the perceived value of the 14 data elements currently contained in the repository (eg, strength, dose, and therapeutic class) and their perceptions of the overall value and impact of the DHDR. Finally, the participants ranked the importance of enhancing the DHDR with specific questions currently under consideration for integration: (1) the comprehensive inclusion of all prescribed medications (ie, the addition of those that are not dispensed), (2) privately paid drugs (ie, private insurance or out-of-pocket claims), and (3) additional clinical data (eg, tolerances or allergies). Nonusers of the DHDR were asked to rate their perceptions of the value of centralized repository access and its potential impact. Further questions using open textboxes were used to elicit the reasons why participants did not use the DHDR and the resources used to develop a BPMH.

Survey items were rated on a 7-point adjectival scale with anchors ranging from *1* (*strongly disagree*) to *7* (*strongly agree*) or *1* (*not at all important/valuable*) to *7* (*extremely important/valuable*). The survey was administered and managed on the web using the Research Electronic Data Capture (REDCap) tool hosted at WCH, in Toronto, Ontario [27,28]. It is a secure web-based software platform designed to support data capture for research studies. Participants who completed the survey had the opportunity to enter into a draw for 1 of the 3 CAD $100 (US $79.46) gift cards.

#### Semistructured Interviews

Interviews were conducted with users and nonusers of the DHDR by trained qualitative research staff with no prior relationship with the study participants (CS and MP), under the supervision of an experienced qualitative researcher (LD). The interviews focused on mechanisms for accessing the DHDR (ie, type of clinical viewer used), features and functions of the repository, perceptions of the data elements contained in the repository, barriers to or enablers of adoption, and potential impact on clinical workflow and health outcomes (Multimedia Appendix 3). Questions were tailored by user type (users and nonusers of the DHDR) and included a demographic questionnaire. Before conducting interviews, the interview guides were reviewed by an experienced qualitative researcher (LD), a pharmacoepidemiologist (MT), and a researcher (external to the research team with expertise in digital health evaluations). The interview questions were derived directly from the research aims, future features, and functionality of the DHDR. The interviews were conducted in person or over the phone, according to the participant’s preference. The interviews were audio-recorded, transcribed, and anonymized by an independent third party.

### Analysis

#### Cross-sectional Survey

Participants were able to skip questions or end the survey early as the survey did not force a response. These incomplete responses were included in the analysis, resulting in different denominators across different questions, and participant responses were aggregated and descriptively analyzed. Items rated on the 7-point adjectival scale using the anchors *strongly disagree* to *strongly agree* were interpreted as disagreement, neutral, and agreement by consolidating responses. Negative statements in the 4 key domains were reverse-scored, and the overall means were calculated for each domain. All the other survey items were aggregated. Subgroup analyses were conducted to assess the impact of gender and user and nonuser perceptions of the impact and value of the DHDR. Differences in gender and user and nonuser responses were evaluated using the Wilcoxon Rank-Sum test. Survey data are presented as means and SD, and statistical significance was considered at the 0.05 level. All analyses were conducted in the R statistical program using the *ggpubr* and *MASS* packages [29].

#### Semistructured Interviews

Interviews were analyzed using qualitative description, a paradigm that seeks to create an understanding of a phenomenon by accessing the meanings that participants ascribe to it [30,31]. Two coders (CS and MP) independently and inductively coded the first three interviews, after which they met to discuss the data, achieve coding alignment, and establish a codebook. One coder (MP) deductively applied the codebook to the remaining interviews, creating inductive codes as new data emerged. New codes were discussed iteratively (CS and MP), and the second coder (CS) reviewed a random subsample of 3 additional interviews to evaluate consistency. Interviews continued until thematic saturation was achieved (ie, no new themes were identified), at which point 3 additional interviews were conducted to confirm saturation [32]. Two team members (CS and MP) then generated preliminary themes that were discussed with a senior scientist (LD) and a pharmacoepidemiologist (MT). Themes were further refined based on group discussions and finalized once a consensus was achieved. Qualitative data were analyzed using NVivo (version 12 Plus; QSR International) [33].

## Results

### Overview

The survey and interview data were collected between May and August 2019. The results are first presented for surveys, followed by the data obtained from the interviews.

### Survey Results

Of the 161 participants who completed the survey, 80.7% (130) were predominantly female and represented a range of health professions (see Table 1 for demographic characteristics). Approximately 32% (53/161) were not using a provincial viewer for patient information, only 24% (38/161) indicated that they were using the DHDR, and 44% (70/161) indicated they were not using the DHDR. Survey validation findings are detailed in Multimedia Appendix 4 [34-36]. Across the key experience domains, the coefficients were negatively correlated, suggesting that the survey items within each section were slightly convergent; however, the findings were not statistically significant.

**Table 1:** Survey participant demographics (N=161).

Demographic attribute						Values, n (%)
**Sex**						
	Male				30 (18.6)	
	Female				131 (81.3)	
**Age (years)**						
	18-34			52 (32.2)		
	35-49			42 (26)		
	50-64			65 (40.3)		
	≥65			1 (0.6)		
	Not reported			1 (0.6)		
**Health care setting**						
	Acute care		93 (57.7)			
	Primary care		24 (14.9)			
	Community care		34 (21.1)			
	Long-term care		2 (1.2)			
	Other		9 (5.4)			
**Primary occupation**						
	Physician	25 (15.5)				
	Nurse	52 (32.2)				
	Pharmacist	45 (27.9)				
	Allied health professional	9 (5.5)				
	Support personnel	7 (4.2)				
	Other^a^	23 (14.2)				
**Primary source of clinical information**						
	Hospital information system				68 (42.2)	
	Electronic medical record				38 (23.46)	
	Client health and related information system				10 (6.2)	
	Paper records				22 (13.6)	
	Other^b^				21 (13)	
	Not reported				2 (1.8)	
**Provincial viewer**						
	ClinicalConnect	82 (50.9)				
	Connecting Ontario	25 (15.5)				
	None of the above	53 (31.9)				
	Not reported	1 (0.6)				
**Used DHDR^c^**						
	No			123 (76.3)		
	Yes			38 (23.6)		

^a^Primary occupations include pharmacies and medical technicians.

^b^The primary source of clinical information includes Meditech, Medstracker, Kroll information systems, and community pharmacy systems.

^c^DHDR: Digital Health Drug Repository.

#### DHDR Users

Of the 40 users who reported using the DHDR, 29 were female (73%), 25 were working in acute care (62%), and 27 were located in an urban setting (68%; Multimedia Appendix 5). Most had accessed the DHDR within the last 6 months, although the frequency of access varied (Multimedia Appendix 6).

**Figure 1:**
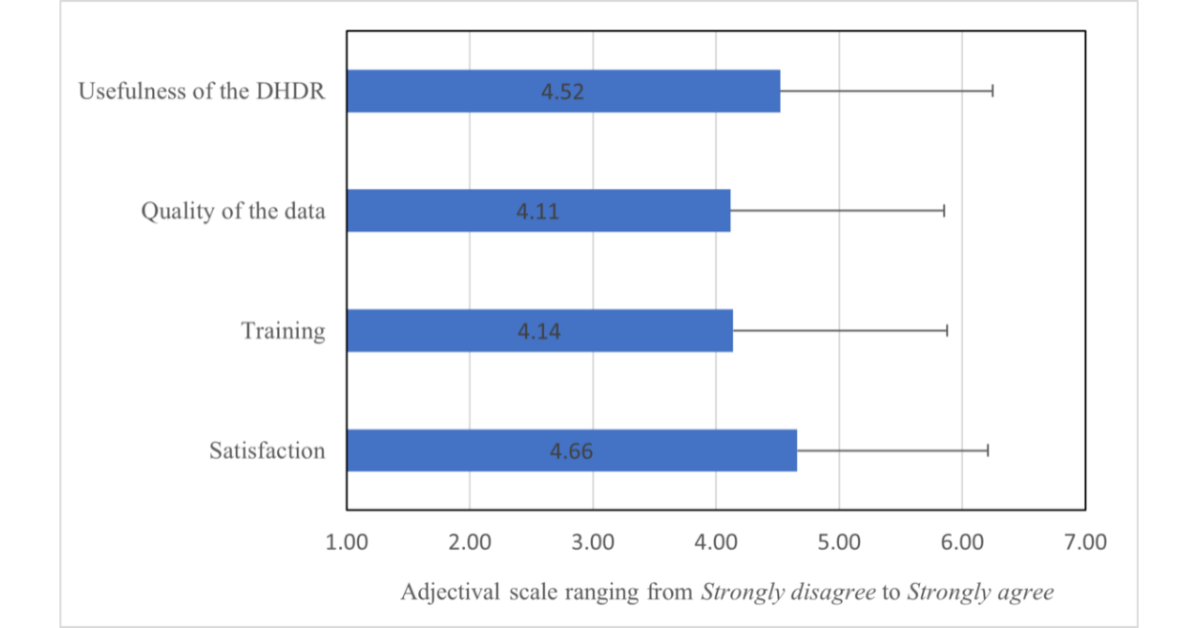
Mean scores and SDs for digital health drug repository (DHDR) experience among survey respondents.

Across all experience domains (ie, usefulness, quality of data, training received, and overall satisfaction), the average participant response trended toward neutral (neither agree nor disagree), with notable variability in responses (Figure 1). Differences were negligible between male and female respondents; however, female respondents (mean score 4.83, SD 1.61) perceived the DHDR to fit with the workflow to a greater degree than their male counterparts (mean score 2.73, SD 1.85; *P*=.003; Multimedia Appendix 7).

Of the 14 data elements currently included in the DHDR (Multimedia Appendix 8), DHDR users rated 9 elements as necessary for the development of a BPMH (with a mean rating ≥5, and SD >1.90). In terms of perceived importance, these included the strength of dose (mean 6.41, SD 0.82), generic name of the drug (mean 6.07, SD 1.24), quantity of medication dispensed (mean 5.97, SD 1.13), prescriber contact information (mean 5.90, SD 1.20), estimated supply (mean 5.87, SD 1.11), dosage form (mean 5.87, SD 1.32), dispense date (mean 5.85, SD 1.14), pharmacy contact information (mean 5.77, SD 1.47), and prescription count (mean 5.45, SD 1.36; Multimedia Appendix 8). Participants expressed a desire for private insurance claims for dispensed medications, medication instructions (eg, 50 mg twice daily), and explicit discontinuations in the DHDR to facilitate a comprehensive profile of medication history.

On average, participants perceived having access to all prescribed medications, dispensed medications, private insurance claims, additional clinical data, and integration of the DHDR into current point-of-care systems as moderately valuable, with average scores ranging from 5.90 to 6.32 (Multimedia Appendix 9). However, participants were neutral in their perceptions of the perceived impact of the DHDR on reducing ADEs, improving patient outcomes, and reducing communication with other providers, with average scores ranging from 4.76 to 4.21 (Multimedia Appendix 10).

#### DHDR Nonusers

A total of 123 participants stated that they were not using the DHDR and had a demographic profile similar to that of the DHDR user group (Multimedia Appendix 5). Most respondents (99/123, 80.5%) had not heard of the DHDR, and 89.4% (110/123) were not familiar with what the DHDR did. Surprisingly, 63.4% (78/123) had access to a provincial viewer, which contained the DHDR, but did not use the DHDR itself (Multimedia Appendix 5). Comments reported in the open textboxes suggest that some DHDR nonusers perceived that there was limited clinically meaningful information in the repository, whereas others believed that the records were incomplete, preferring instead to rely on pharmacy records. Those who did not have access to a provincial viewer cited a lack of availability or the tedious registration process as barriers to use.

#### Comparison of DHDR Users and Nonusers

**Figure 2:**
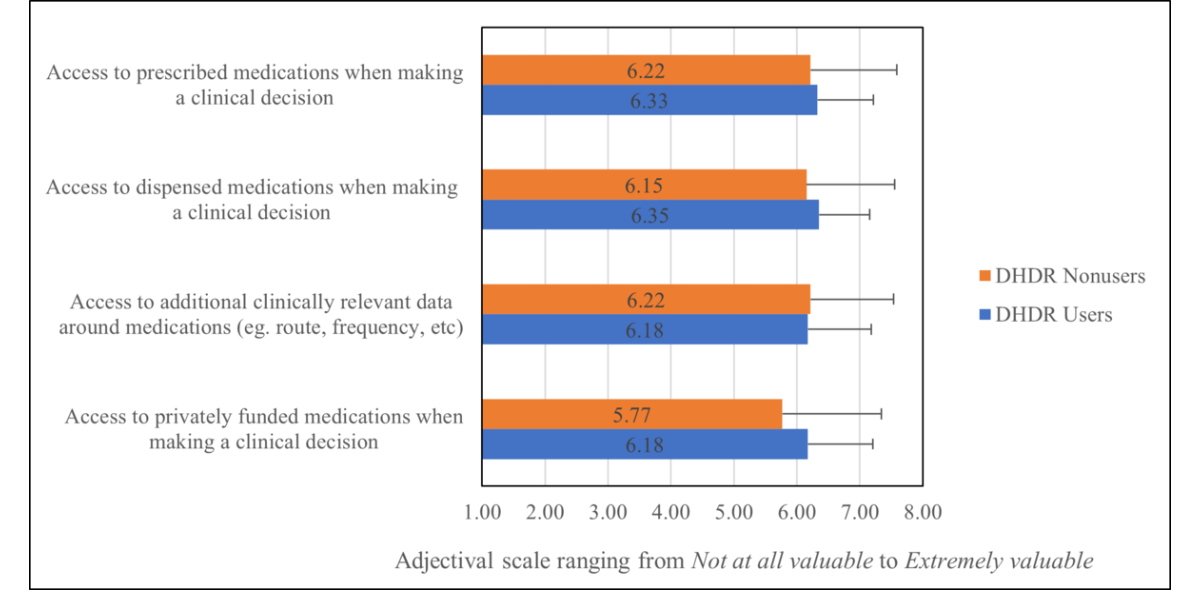
Perceptions of the value of accessing information at the point of care among survey respondents. Bars represent mean scores and error bars represent SDs. DHDR: Digital Health Drug Repository.

**Figure 3:**
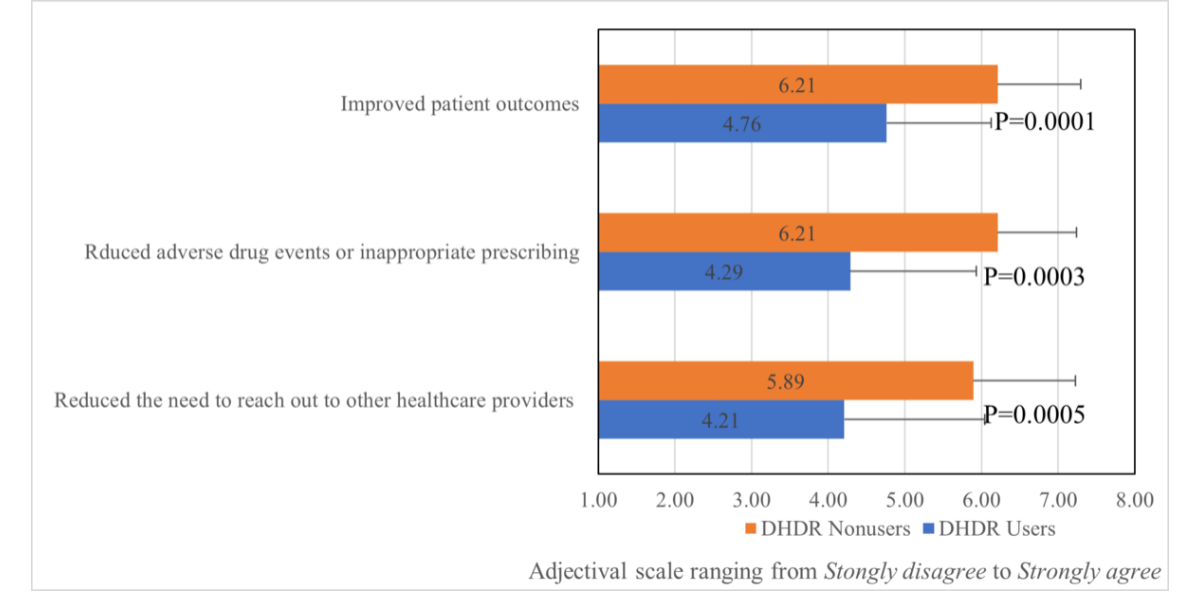
Perceptions of the impact of the digital health drug repository (DHDR) among survey respondents. Bars represent mean scores and error bars represent SDs.

Overall, users and nonusers of the DHDR expressed the value in the ability to access all prescribed medications, dispensed medications, privately funded medications, and additional clinical data at the point of care when developing a BPMH (Figure 2), with no significant differences between the groups. All participants agreed that a centralized repository would improve patient outcomes, reduce unnecessary communication between clinicians, and reduce ADEs (Figure 3).

### Interviews

A total of 33 clinicians were interviewed between May 13 and August 1, 2019. The average length of the interviews was 25 minutes (range 7-53 minutes). Interviewees had a similar demographic profile to that of the survey respondents, apart from a greater gender balance (Table 2). Of the 33 participants, 26 (79%) had access to a provincial viewer and had accessed the DHDR at least once, and 7 (21%) participants neither had access nor wanted to pursue the process to get access because they believed that they were ineligible for access or were concerned about the administrative process to obtain access. Moreover, nonusers were generally satisfied with current resources, such as calling pharmacists or health care provider to obtain medication information. Themes centered around user experience, training and onboarding, resources used for BPMH, clinical use cases, impact on clinical workflow, lack of awareness of the DHDR, and perceived impact. Additional quotes supporting key themes can be found in Multimedia Appendix 11.

**Table 2:** Demographics of interviewed participants (N=33).

Demographic attribute		Participants, n (%)						
**Gender**								
	Women		20 (61)					
	Men		13 (39)					
**Location**								
	Urban				23 (70)			
	Rural				6 (18)			
	Both				4 (12)			
**Health care setting**								
	Acute care		15 (46)					
	Community care			10 (33)				
	Primary care				5 (15)			
	Long-term care				1 (3)			
	Other^a^				2 (7)			
**Primary occupation**								
	Pharmacist					15 (46)		
	Physician					13 (39)		
	Other^b^					5 (15)		
**Provincial clinical viewer**								
	Clinical connect					13 (39)		
	Connecting Ontario					13 (39)		
	No access					7 (21)		

^a^Multiple settings (ie, acute and primary care) in the health care setting.

^b^Includes occupational therapists, pharmacy technicians, medical laboratories, and ultrasound technologists in a primary occupation.

#### User Experience

Overall, 26 participants accessed the repository a few times a month and were generally satisfied with the available data. Participants found the DHDR easy to use and adaptable to their clinical needs (eg, the ability to filter medication histories over a customizable time). Clinicians valued the basic medication information contained in the repository such as drug name, dosage form, and contact information of the prescriber and dispensing pharmacy as secondary mechanisms to validate BPMH.

Participants felt that the DHDR did not fully achieve its intended role as it did not capture all medication information for Ontario residents (ie, only those covered), restricting its ability to provide a complete and comprehensive understanding of a patient’s medication history. Relatedly, notable information was missing from the DHDR, including medication instructions, alternative medication names, drug discontinuations, a comprehensive list of prescribed medications (ie, including those that were not dispensed), and private insurance claims for dispensed medications. None of the participants used the DHDR as the primary source of medication information and only used when it was not possible to call the pharmacy or family physician (ie, after hours). Methods that clinicians used to conduct a BPMH included reviewing the patient’s health record, conducting a patient interview, confirming medications with a pharmacist or family physician, and using other resources such as electronic medical records.

Participants were able to identify issues with medications, such as potential drug interactions, ADEs, or duplicate prescriptions because of the DHDR. Some participants expressed that not having access to this information generates unnecessary costs to the health care system because of duplicate prescriptions and risk of complications.

#### Onboarding and Training

Obtaining access to provincial viewers (within which the DHDR is contained) presented challenges for participants working in community or long-term care settings. Those working in acute care settings gained access through their organization and delays were relatively small (ie, a few days to weeks). For those outside of acute care, the process of obtaining access was tedious and lengthy, with some participants reporting waiting periods to gain access ranging from 2.5 to 18 months. Delays were often the result of security and privacy assessments or waiting for a response to inquiries during registration. The process of obtaining access to the DHDR for a large hospital was the same as that for a small community practice or a local pharmacy, and participants were often frustrated at the process. Out-of-pocket overhead costs and resources required to achieve provincial viewer access were a concern to community providers, which served as a deterrent to some:

You basically go on the website and then you look at the link that talks about getting access for your clinic. Then you contact somebody and they usually will take their time to get back to you, but within maybe a month they'll get back to you and they'll put you on the waiting list and you'll wait another month and then they'll call you back to arrange a time. Kind of works like that. Then, they'll come to your clinic to give you a little bit of orientation and then they'll set it up.

P5

#### Clinical Use Cases

Participants agreed that having access to comprehensive medication information at the point of care is valuable for clinical decision-making. Access to medication information was highlighted as particularly useful when conducting a BPMH for geriatric patients, complex patients, and those at high risk for readmission (eg, patients with chronic obstructive pulmonary disorder). The DHDR was particularly valuable in emergency departments at night and during surgical consultations when the ability to contact pharmacies and family physicians was either not possible or limited. Among family physicians, the DHDR was primarily used to support clinical decisions related to antibiotic or narcotic prescriptions for new patients.

#### Factors Limiting Impact

At times, clinicians with access to the DHDR found integration into their clinical workflow challenging. Inefficiencies were created by the need to log out of their usual point-of-care system to log on to the provincial viewer to access the repository. Both DHDR users and nonusers expressed a desire to have centralized repositories integrated within their point-of-care systems to minimize disruptions to the workflow:

As you can imagine, somebody...is doing a consult, they're opening up their computer, they're looking through [their EMR]...But then, in order to get to ConnectingOntario you have to actually open up a different window...you actually have to come out of [the EMR] to load up another window, which takes you away from what you were doing before...That takes time. It takes time to load. So, I think you should...actually integrate it into an EMR system, so that the information can be accessed easily instead of through the ConnectingOntario interface.

P5

Nonusers of the DHDR were unaware of how to register for access to the DHDR, and some participants in community settings highlighted the financial and administrative burden of gaining access to a provincial viewer. Others were unaware of the process of obtaining access, and some community physicians and pharmacists were unclear if they were eligible for access as they worked in independent practices.

## Discussion

### Principal Findings

To the best of our knowledge, this is the first evaluation of the DHDR repository in Ontario. Previous evaluations of similar national and international repositories have focused on the quality of data contained in the repository and how this affected clinical and process outcomes (eg, inappropriate prescribing, accuracy, and completeness of data elements, medication profiles, and compliance with BPMH documentation) [14,37,38]. Several evaluation studies found drug repositories to be incomplete and contain several discrepancies, thereby requiring use in conjunction with other sources to validate medication histories, and as such, drug repositories may be underused [14,39].

Our results highlight the untapped potential of the DHDR and provide insights into the key elements that are likely to drive future clinical value. Specifically, clinicians found the DHDR valuable in clinical situations where prompt communication with other clinicians or the pharmacy was not feasible, such as in the emergency department. A general lack of awareness was a significant barrier to realizing value at the system level, whereas a lack of comprehensive data was a barrier to consistent use. Moreover, the lack of integration into existing point-of-care systems was highlighted as a challenge to usage. Expanding the DHDR data set to include information on medication instructions and private insurance claims would enhance the clinical value and relative advantage of the DHDR.

The DHDR was most meaningful for clinicians who care for complex or older patients, such as geriatricians, underscoring the value of a comprehensive profile. Most of these patient populations are likely to be supported through publicly funded programs in which complete medication histories are captured within the DHDR. This suggests a potential use case in long-term care facilities, where polypharmacy is a common problem and the prevalence of ADEs is higher than that in other health care settings (ranging from 18% to 82%) [40]. Polypharmacy also increases the likelihood of hospital admission for patients living in long-term care [41], further emphasizing the potential untapped value of a centralized drug repository. Our sample had minimal representation from long-term care facilities, highlighting the need to further explore how to create value for clinicians working in this sector. The lack of medication information for most Ontario residents precluded meaningful use in most other settings. Only 20% of Ontario’s 14.6 million residents are covered by public drug programs [22,42], limiting the DHDR’s ability to deliver value for most of the population. Inclusion of all prescribed medications (whether dispensed) and the integration of community pharmacy records would support increased medication adherence and the reduction of inappropriate prescribing [43,44].

Despite a clear pathway to comprehensive data, a lack of awareness of the DHDR presents a fundamental barrier to achieving an impact at the population level. Although many participants perceived the data elements to be valuable, they were unaware that the repository existed or had limited knowledge on how to obtain access. This problem is not unique to the DHDR [45] and highlights the need for active dissemination strategies, such as engaging local clinical champions and increasing awareness and education about the repository through professional organizations (eg, the Ontario Medical Association, the Registered Nurses Association of Ontario, or the Ontario College of Pharmacists), which may increase the uptake [46-48]. Cumbersome access pathways are not unique to the DHDR [45], suggesting that efforts to streamline onboarding, such as bundling it directly to licensure or credentialing processes rather than keeping it as an independent activity, would have positive downstream impacts beyond those realized through the DHDR. Integration into existing point-of-care systems is one such evidence-based strategy that would overcome a key barrier to adoption that plagues a range of digital technologies in health care [39,45,49,50] and has been successfully operationalized for a centralized drug repository [20].

### Limitations

This formative evaluation aimed to capture responses from clinicians across multiple health care settings, as well as a broad sample of users and nonusers, to highlight areas for enhancements to the DHDR, which may increase meaningful clinical use. Consequently, our findings cannot speak to the realization of the impact on patient outcomes, experience, and system cost. Our findings are specific to sites with ≥20 registered users and may not reflect the experiences of providers in smaller centers (eg, rural communities). Although more female clinicians participated in our study, we feel this is comparable with the Ontario health care workforce, with over 70% being women. Moreover, we did not know how many individuals were sent the survey link, and as such, our findings may have selection bias because of voluntary self-selection.

As the survey was developed primarily by our project partners to inform decision-making, we sought to evaluate face validity and potential correlation across key domains (Multimedia Appendix 4). No statistical significance was observed, may be because of the limited number of active DHDR users, underscoring the need for further and more robust validity testing.

We were limited in our ability to assess the impact of the perceived shortcomings of the DHDR and associated clinical viewers. Teasing apart users’ perceptions of the DHDR from their clinical viewer experience was beyond the scope of the project; however, it is important to acknowledge. The DHDR was not co-designed by a representative sample of those using the repository, and only contained medication information for a subset of the Ontario population, which limited its clinical value. It is important to note that the DHDR was purposively implemented as an *incomplete* product by eHealth to facilitate the ability to gather data and insights to inform future development and expansion. Limited uptake suggests that future development efforts must consider marketing and implementation efforts to realize the impact. Finally, our objective was to understand health care provider perceptions, usage, and the clinical value of the DHDR to inform strategies to increase its utility and uptake. We determined that the uptake of the DHDR is a necessary precursor to its potential impact on care. Although patient experience is a critical next step in understanding whether and how such a tool can have an impact, it was beyond the scope of this project. An important evolution of this study is understanding how access to comprehensive medication data is valued by patients and how it can be used to enhance patient experience.

### Conclusions

Findings from this formative evaluation suggest that the DHDR has untapped value as currently operationalized but that a pathway exists to align with clinician needs. Ensuring comprehensive clinical data and streamlined onboarding processes would facilitate meaningful use, whereas integration with existing point-of-care systems would further enhance efficiency and uptake. Access to a centralized repository that connects currently fragmented health care settings and provides comprehensive medication information at the point of care has the potential to improve efficiency and reduce medication errors if it aligns with the informational needs of clinical decision-making. Stakeholders involved in operationalizing and implementing such repositories (or similar information-sharing systems) should consider broad marketing and dissemination, user engagement, and integration into existing workflows as part of their overall strategy to realize value. Finally, user confidence in the DHDR can be improved by validating the information contained in the repository by comparing multiple sources of BPMH (ie, patient interviews vs pharmacy records vs DHDR records) and documenting the number of medication omissions, inappropriate prescribing, and discrepancies to validate DHDR. The knowledge that DHDR is accurate and complete will increase the uptake.

## Appendix

Multimedia Appendix 1 Clinical Sensibility Questionnaire.

Multimedia Appendix 2 Survey questions.

Multimedia Appendix 3 Interview guide.

Multimedia Appendix 4 Survey validation results for key experience domains.

Multimedia Appendix 5 Comparison of demographic information in the survey for digital health drug repository (DHDR) users and nonusers (N=161).

Multimedia Appendix 6 Frequency of digital health drug repository (DHDR) use among survey respondents (N=40).

Multimedia Appendix 7 Mean scores and SDs of the digital health drug repository experience domains among survey respondents.

Multimedia Appendix 8 Mean scores and SDs of the usefulness of current digital health drug repository (DHDR) data elements among DHDR users in the survey.

Multimedia Appendix 9 Rankings for new digital health drug repository (DHDR) data elements among survey respondents.

Multimedia Appendix 10 Overall perceptions of the value and impact of the digital health drug repository (DHDR) among female and male DHDR users (N=40).

Multimedia Appendix 11 Outstanding quotes.

## Abbreviations

ADE: adverse drug event
BPMH: best possible medication history
DHDR: Digital Health Drug Repository
LRA: Local Registration Authority
