# Supplementary material for: Understanding Engagement and the Potential Impact of an Electronic Drug Repository: Multi-Methods Study
Source: JMIR Form Res. 2022 Mar 30;6(3):e27158. doi: 10.2196/27158 (PMC9008523; doi:10.2196/27158)
Supplement: Multimedia Appendix 1 [file formative_v6i3e27158_app1.docx]

**Appendix 1. Clinical Sensibility Questionnaire**

Dr. Laura Desveaux and the research team are working on an evaluation of the Digital Health Drug Repository (DHDR) and request your help in assessing the clinical sensibility of a survey. Please answer the following questions:

1. To what extent are **the questions** on the DHDR survey simple and easy to understand?

Small

Extent

Limited

Extent

Fair

Extent

Large

Extent

Moderate

Extent

1. To what extent are the **response options** provided on the DHDR survey simple and easy to understand?

Small

Extent

Fair

Extent

Large

Extent

Moderate

Extent

Limited

Extent

1. How many **items** on the DHDR survey are inappropriate or redundant?

Very Many

Many

Some

A few

None

Please i

1. How long did it take you to complete the DHDR survey? _______ minutes

Please feel free to leave additional comments or feedback in the box below.

Thank you for assisting us with the sensibility testing of our DHDR survey!

**Adapted from:** Burns et al. A guide for the design and conduct of self-administered survey of clinicians. CMAJ 2008; 179(3):245-252.
