# Supplementary material for: Understanding Engagement and the Potential Impact of an Electronic Drug Repository: Multi-Methods Study
Source: JMIR Form Res. 2022 Mar 30;6(3):e27158. doi: 10.2196/27158 (PMC9008523; doi:10.2196/27158)
Supplement: Multimedia Appendix 2 [file formative_v6i3e27158_app2.docx]

# **Appendix 2. Survey questions.**

**Demographic questions**

| 1. I am: | | | *Allied Health Professional* | |
| --- | --- | --- | --- | --- |
|  Male | |  Non-binary |  Pharmacist |  Physiotherapist |
|  Female | |  |  Midwife |  Nutritionist/Dietician |
|  | | |  Psychologist |  Respiratory therapist |
| 2. Please select your age category: | | |  Social worker |  |
|  18-34 years old | |  50-64 years old |  | |
|  35-49 years old | |  ≥ 65 years old | *Support Personnel* | |
|  | | |  Case Manager |  Care Coordinator |
| 3. Which area of health care setting do you primarily work in? | | |  | |
|  Acute care | |  Long term care | *Administrative* | |
|  Primary care | |  Other (please specify): |  Clerk | |
|  Community care | | __________________________ |  | |
|  | | | *Other Personnel* | |
| <<If **acute care**is selected>> | | |  Other (please specify):___________________________ | |
| 3.a. Please select the specific area in which you primarily work: | | |  | |
|  Emergency department | | | 6. What patient record system do you use as your primary | |
|  Intensive care unit | |  Surgical unit | source of clinical information? | |
|  General medical unit | |  Other (please specify): |  Hospital information |  Paper records |
|  Specialist medical unit | | __________________________ | system |  Other (please specify): |
| (i.e. oncology, orthopedic, | |  |  Ontario MD approved | ______________________ |
| cardiology, geriatrics) | |  | Primary Care Electronic |  |
|  | | | Medical Records (EMR) |  |
| <<If **primary care**is selected>> | | |  Client Health and |  |
| 3.b. Please select the specific area in which you primarily work: | | | Related Information |  |
|  Family Health Group | |  Other (please specify): | System (CHRIS) |  |
|  Solo practice | | __________________________ |  | |
|  Family Health Network | |  | 7. Which provincial viewer do you primarily use to access | |
|  | | | the DHDR (also referred to as the medications portlet, | |
| <<If **community care**is selected>> | | | home meds or the pharmacy module in your clinical | |
| 3.c. Please select the specific area in which you primarily work: | | | viewer)? | |
|  Pharmacy | |  Other (please specify): |  ClinicalConnect |  ConnectingOntario |
|  Mental health facility | | __________________________ |  | |
|  Community health centre | |  | 8. How long have you had access to the DHDR? | |
|  | | |  ≤ 3 months | |
| <<If **long term care**is selected>> | |  |  3-6 months | |
| 3.d. Please select the specific area in which you primarily work: | | |  6-12 months | |
|  Nursing home |  Other (please specify): | |  ≥ 12 months | |
|  Retirement home | __________________________ | |  | |
|  Assisted living facility |  | | 9. On average, how many times do you access the DHDR | |
|  | | | in a month? | |
| 4. How would you characterize the health care setting in which you | | |  0-4 times | |
| work? | | |  5-9 times | |
|  Urban | |  Other (please specify): |  10-14 times | |
|  Rural | | __________________________ |  15-19 times | |
|  | | |  ≥20 times | |
| 5. Which of the following best represents your primary occupation | | |  | |
| (Please select one) | | |  | |
| *Medicine* | | |  | |
|  Specialist physician |  Primary care physician | |  | |
|  Emergency physician |  | |  | |
|  | | |  | |
| *Nursing* | | |  | |
|  Nurse practitioner |  Registered Practical Nurse | |  | |
|  Registered Nurse |  | |  | |

**Survey Questions**

Please select the choice that reflects how you feel regarding your use of the DHDR.

**Usefulness of DHDR**

1. Constructing my patient's medication history using the information provided in the DHDR is difficult.

Strongly disagree Strongly agree

1 2 3 4 5 6 7

1. Using the DHDR saves time when developing a best possible medication history.

Strongly disagree Strongly agree

1 2 3 4 5 6 7

1. The DHDR contains the features/information that I need to conduct a best possible medication history

Strongly disagree Strongly agree

1 2 3 4 5 6 7

1. The DHDR fits well within my clinical routine/workflow

Strongly disagree Strongly agree

1 2 3 4 5 6 7

**Quality of data in DHDR**

1. The DHDR does not contains the right data to establish a patient's medication history

Strongly disagree Strongly agree

1 2 3 4 5 6 7

1. The DHDR helps identify potentially harmful drug interactions/reactions

Strongly disagree Strongly agree

1 2 3 4 5 6 7

1. The DHDR provides timely information on a patient's dispensed medication history

Strongly disagree Strongly agree

1 2 3 4 5 6 7

1. The DHDR shortens the medical and/or nursing assessment time

Strongly disagree Strongly agree

1 2 3 4 5 6 7

**Implementation/Training**

1. The process to obtain access to the DHDR was simple and straightforward

Strongly disagree Strongly agree

1 2 3 4 5 6 7

1. I obtained access to the DHDR in a timely manner

Strongly disagree Strongly agree

1 2 3 4 5 6 7

1. The training and materials I received on the DHDR was sufficient and easy to understand

Strongly disagree Strongly agree

1 2 3 4 5 6 7

1. I often encounter technical issues when accessing the DHDR

Strongly disagree Strongly agree

1 2 3 4 5 6 7

**Overall satisfaction**

1. Access to the DHDR allows me to improve the quality of care I provide

Strongly disagree Strongly agree

1 2 3 4 5 6 7

1. The DHDR has enhanced my ability to coordinate continued care

Strongly disagree Strongly agree

1 2 3 4 5 6 7

1. The DHDR is confusing to use and navigate

Strongly disagree Strongly agree

1 2 3 4 5 6 7

1. Overall, I am satisfied with the DHDR

Strongly disagree Strongly agree

1 2 3 4 5 6 7

**Perception**

1. Do you feel that having access to DHDR reduced the need to reach out to other healthcare providers when making a clinical decision/ developing a best possible medication history?

Strongly disagree Strongly agree

1 2 3 4 5 6 7

1. Do you feel that having access to DHDR reduces adverse drug events or inappropriate prescribing?

Strongly disagree Strongly agree

1 2 3 4 5 6 7

1. Do you feel that having access to DHDR improves patient outcomes?

Strongly disagree Strongly agree

1 2 3 4 5 6 7

**Usefulness of current data elements**

1. Please indicate how important the following items are when making a clinical decision/developing a best possible medication history.

Dispensing Information

- Dispensed date (When the prescription was dispensed or the pharmacy service was rendered)

Not at all important Extremely important

|  | 1 | 2 | 3 | 4 | 5 | 6 | 7 |
| --- | --- | --- | --- | --- | --- | --- | --- |

- Prescription count (The numerical count of the dispensed drug or pharmacy service events in each grouping)

Not at all important Extremely important

|  | 1 | 2 | 3 | 4 | 5 | 6 | 7 |
| --- | --- | --- | --- | --- | --- | --- | --- |

- Quantity of medication dispensed

Not at all important Extremely important

|  | 1 | 2 | 3 | 4 | 5 | 6 | 7 |
| --- | --- | --- | --- | --- | --- | --- | --- |

- Pharmacy contact information

Not at all important Extremely important

|  | 1 | 2 | 3 | 4 | 5 | 6 | 7 |
| --- | --- | --- | --- | --- | --- | --- | --- |

Drug Information

- Generic name (Non-proprietary name of the drug dispensed). For Pharmacy Services, this field will contain the description of the pharmacy service rendered by a pharmacist.

Not at all important Extremely important

|  | 1 | 2 | 3 | 4 | 5 | 6 | 7 |
| --- | --- | --- | --- | --- | --- | --- | --- |

- Brand name (Trade name of the prescription drug dispensed. For Pharmacy Services, this field will contain the pharmacy service type).

Not at all important Extremely important

|  | 1 | 2 | 3 | 4 | 5 | 6 | 7 |
| --- | --- | --- | --- | --- | --- | --- | --- |

- Strength (Amount of active ingredient in the prescription drug dispensed)

Not at all important Extremely important

|  | 1 | 2 | 3 | 4 | 5 | 6 | 7 |
| --- | --- | --- | --- | --- | --- | --- | --- |

- Dosage form (The physical form of a dose of a drug product)

Not at all important Extremely important

|  | 1 | 2 | 3 | 4 | 5 | 6 | 7 |
| --- | --- | --- | --- | --- | --- | --- | --- |

- Therapeutic class (e.g., analgesics nonsteroidal))

Not at all important Extremely important

|  | 1 | 2 | 3 | 4 | 5 | 6 | 7 |
| --- | --- | --- | --- | --- | --- | --- | --- |

- Therapeutic subclass(e.g. anti-inflammatory agent))

Not at all important Extremely important

|  | 1 | 2 | 3 | 4 | 5 | 6 | 7 |
| --- | --- | --- | --- | --- | --- | --- | --- |

Other Information

- Prescriber contact information

Not at all important Extremely important

|  | 1 | 2 | 3 | 4 | 5 | 6 | 7 |
| --- | --- | --- | --- | --- | --- | --- | --- |

- Estimated supply (Estimated number of days of treatment based on the directions for use on the prescription and/or the pharmacist's judgment on usage)

Not at all important Extremely important

|  | 1 | 2 | 3 | 4 | 5 | 6 | 7 |
| --- | --- | --- | --- | --- | --- | --- | --- |

- Drug Utilization Review (Historical Information on drug alerts that were generated and communicated to the dispenser when a drug claim was submitted for adjudication ("approval") or when a monitored drug dispense event record is submitted to the Narcotics Monitoring System, and include drug to drug interactions). .

Not at all important Extremely important

|  |  | |  | |  | |  | |  | |  | |  | |  |
| --- | --- | --- | --- | --- | --- | --- | --- | --- | --- | --- | --- | --- | --- | --- | --- |
|  | | 1 | | 2 | | 3 | | 4 | | 5 | | 6 | | 7 | |

- - DIN (drug information number assigned by Health Canada to a drug product marketed in Canada)

Not at all important Extremely important

|  |  | |  | |  | |  | |  | |  | |  | |  |
| --- | --- | --- | --- | --- | --- | --- | --- | --- | --- | --- | --- | --- | --- | --- | --- |
|  | | 1 | | 2 | | 3 | | 4 | | 5 | | 6 | | 7 | |

1. Are there other features or data elements that you think should be included in the repository? Please comment below.

**Value added**

1. How valuable is it to have access to prescribed medications when making a clinical decision/ developing a best possible medication history?

Not at all valuable Extremely valuable

|  | 1 | 2 | 3 | 4 | 5 | 6 | 7 |
| --- | --- | --- | --- | --- | --- | --- | --- |

1. How valuable is it to have access to dispensed medications when making a clinical decision/ developing a best possible medication history?

Not at all valuable Extremely valuable

|  | 1 | 2 | 3 | 4 | 5 | 6 | 7 |
| --- | --- | --- | --- | --- | --- | --- | --- |

1. How valuable is it to have access to additional clinically relevant data elements (e.g. Route, Frequency, Dose Instructions, etc.) when making a clinical decision/ developing a best possible medication history?

Not at all valuable Extremely valuable

|  | 1 | 2 | 3 | 4 | 5 | 6 | 7 |
| --- | --- | --- | --- | --- | --- | --- | --- |

1. How valuable is it to access privately funded medications when making a clinical decision/developing a best possible medication history?

Not at all valuable Extremely valuable

|  | 1 | 2 | 3 | 4 | 5 | 6 | 7 |
| --- | --- | --- | --- | --- | --- | --- | --- |

1. How valuable is it to have DHDR integration into your hospital information system or electronic medical record when making a clinical decision/developing a best possible medication history?

Not at all valuable Extremely valuable

|  | 1 | 2 | 3 | 4 | 5 | 6 | 7 |
| --- | --- | --- | --- | --- | --- | --- | --- |

1. How valuable is it to have DHDR used more broadly to facilitate communication with healthcare providers?

Not at all valuable Extremely valuable

|  | 1 | 2 | 3 | 4 | 5 | 6 | 7 |
| --- | --- | --- | --- | --- | --- | --- | --- |

1. For the greatest impact on patient care, please prioritize the following with (1) being the most important to (4) being the least important.
2. Including Prescribed Medications in the DHDR
3. Including Additional Clinically Relevant Data Elements in the DHDR
4. Including Privately Paid Medications in the DHDR
5. Increasing deployment of the existing DHDR (in the absence of enhancements a, b, or c) to a broader user group

<<if NO to question 8 (Non-DHDR users)>>

1. Have you heard of the Digital health drug repository (DHDR)?

- YES
- NO

1. Are you familiar with what the DHDR does?

- YES
- NO

<< if yes>>

1. What are the reasons for not using DHDR?
2. What resources do you use to make a best possible medication history?
3. Have you ever used the Drug Profile Viewer (DPV)

- YES
- NO

<<if YES, 3-4>>

1. Why do you prefer the DPV
2. Is there information that you would like access to but currently do not have in DPV?
3. How valuable is it to have access to prescribed medications when making a clinical decision/ developing a best possible medication history?

Not at all valuable Extremely valuable

|  | 1 | 2 | 3 | 4 | 5 | 6 | 7 |
| --- | --- | --- | --- | --- | --- | --- | --- |

1. How valuable is it to have access to dispensed medications when making a clinical decision/ developing a best possible medication history?

Not at all valuable Extremely valuable

|  | 1 | 2 | 3 | 4 | 5 | 6 | 7 |
| --- | --- | --- | --- | --- | --- | --- | --- |

1. How valuable is it to have access to clinically relevant data elements around medications (e.g. Route, Frequency, Dose Instructions, etc.) when making a clinical decision/ developing a best possible medication history?

Not at all valuable Extremely valuable

|  | 1 | 2 | 3 | 4 | 5 | 6 | 7 |
| --- | --- | --- | --- | --- | --- | --- | --- |

1. How valuable is it to access privately funded medications when making a clinical decision/developing a best possible medication history?

Not at all valuable Extremely valuable

|  | 1 | 2 | 3 | 4 | 5 | 6 | 7 |
| --- | --- | --- | --- | --- | --- | --- | --- |

1. Do you feel that having access to a patient's medication history would reduced the need to reach out to other healthcare providers when making a clinical decision/ developing a best possible medication history?

Strongly disagree Strongly agree

1 2 3 4 5 6 7

1. Do you feel that having access to a patient's medication history would reduce adverse drug events or inappropriate prescribing?

Strongly disagree Strongly agree

1 2 3 4 5 6 7

1. Do you feel that having access to a patient's medication history would improve patient outcomes?

Strongly disagree Strongly agree

1 2 3 4 5 6 7
