# Supplementary material for: Understanding Engagement and the Potential Impact of an Electronic Drug Repository: Multi-Methods Study
Source: JMIR Form Res. 2022 Mar 30;6(3):e27158. doi: 10.2196/27158 (PMC9008523; doi:10.2196/27158)
Supplement: Multimedia Appendix 3 [file formative_v6i3e27158_app3.docx]

# **Appendix 3. Interview guide.**

**Demographic questions**

| 1. I am: | | | *Allied Health Professional* | |
| --- | --- | --- | --- | --- |
|  Male | |  Non-binary |  Pharmacist |  Physiotherapist |
|  Female | |  |  Midwife |  Nutritionist/Dietician |
|  | | |  Psychologist |  Respiratory therapist |
| 2. Please select your age category: | | |  Social worker |  |
|  18-34 years old | |  50-64 years old |  | |
|  35-49 years old | |  ≥ 65 years old | *Support Personnel* | |
|  | | |  Case Manager |  Care Coordinator |
| 3. Which area of health care setting do you primarily work in? | | |  | |
|  Acute care | |  Long term care | *Administrative* | |
|  Primary care | |  Other (please specify): |  Clerk | |
|  Community care | | __________________________ |  | |
|  | | | *Other Personnel* | |
| <<If **acute care**is selected>> | | |  Other (please specify):___________________________ | |
| 3.a. Please select the specific area in which you primarily work: | | |  | |
|  Emergency department | | | 6. What patient record system do you use as your primary | |
|  Intensive care unit | |  Surgical unit | source of clinical information? | |
|  General medical unit | |  Other (please specify): |  Hospital information |  Paper records |
|  Specialist medical unit | | __________________________ | system |  Other (please specify): |
| (i.e. oncology, orthopedic, | |  |  Ontario MD approved | ______________________ |
| cardiology, geriatrics) | |  | Primary Care Electronic |  |
|  | | | Medical Records (EMR) |  |
| <<If **primary care**is selected>> | | |  Client Health and |  |
| 3.b. Please select the specific area in which you primarily work: | | | Related Information |  |
|  Family Health Group | |  Other (please specify): | System (CHRIS) |  |
|  Solo practice | | __________________________ |  | |
|  Family Health Network | |  | 7. Which provincial viewer do you primarily use to access | |
|  | | | the DHDR (also referred to as the medications portlet, | |
| <<If **community care**is selected>> | | | home meds or the pharmacy module in your clinical | |
| 3.c. Please select the specific area in which you primarily work: | | | viewer)? | |
|  Pharmacy | |  Other (please specify): |  ClinicalConnect |  ConnectingOntario |
|  Mental health facility | | __________________________ |  | |
|  Community health centre | |  | 8. How long have you had access to the DHDR? | |
|  | | |  ≤ 3 months | |
| <<If **long term care**is selected>> | |  |  3-6 months | |
| 3.d. Please select the specific area in which you primarily work: | | |  6-12 months | |
|  Nursing home |  Other (please specify): | |  ≥ 12 months | |
|  Retirement home | __________________________ | |  | |
|  Assisted living facility |  | | 9. On average, how many times do you access the DHDR | |
|  | | | in a month? | |
| 4. How would you characterize the health care setting in which you | | |  0-4 times | |
| work? | | |  5-9 times | |
|  Urban | |  Other (please specify): |  10-14 times | |
|  Rural | | __________________________ |  15-19 times | |
|  | | |  ≥20 times | |
| 5. Which of the following best represents your primary occupation | | |  | |
| (Please select one) | | |  | |
| *Medicine* | | |  | |
|  Specialist physician |  Primary care physician | |  | |
|  Emergency physician |  | |  | |
|  | | |  | |
| *Nursing* | | |  | |
|  Nurse practitioner |  Registered Practical Nurse | |  | |
|  Registered Nurse |  | |  | |

**Interview Questions**

1. Who are the patients that you would conduct a best possible medication history for?
   1. Are there particular types of patients or particular health care settings?
2. How well are you able to determine a best possible medication history for your patients?
   1. Can you give me an example of how this information factors into your clinical decision making?
3. What resources do you use when determining a best possible medication history for a patient?
   1. Why do you prefer those resources?
   2. Is there information you would like to have but can't access?
4. How valuable is having access to a patient's medication history versus other clinical information (probe for examples)?
5. Are you aware of the DHDR? << questions 5-13 if they state YES to this question>>
   1. Which viewer do you use?
   2. How did you gain access to the DHDR? Can you describe your onboarding experience/training?
   3. Can you describe the consent process in DHDR?
      1. What do you do when a participant has placed a block on their medication history?
   4. How satisfied are you with this process? Can you describe any delays/challenges/frustrations you experience when it comes to accessing the DHDR?
6. What information do you value in the DHDR?
7. What information do you use in DHDR to make a best possible medication history?
   1. What additional information would you like to see in DHDR to help inform best possible medication history?
   2. Are there any features you would like to see in the repository that don't currently exist?
8. Can you walk me through an example of how the DHDR fits (or doesn't fit) into your clinical workflow?
   1. What settings would you use the DHDR (e.g., emerg, ambulatory clinic, OR ICU)?
   2. Are there any differences between the different health care settings?
9. What are your perceived benefits of having access to dispensed drug information? OR can you describe a situation in which the DHDR had value for you?
   1. Which settings do you think the DHDR would be most appropriate for? Who else could benefit from access to DHDR?
10. What are the current barriers to adoption of the DHDR?
    1. What should we do to get more people to use/be aware of the DHDR
11. To what extent does your ability to access the DHDR impact on the timeliness with which you can provide care? How?
12. How easy is it to construct your patient's medication history using the information provided in the DHDR?
    1. Are there any data that are hard to interpret?
13. Are you aware of what medications are NOT listed in the DHDR?
    1. How do you access that missing data specifically?
14. Why do you choose not to use the DHDR?
    1. Are there specific factors that make it difficult to use? (probe – navigating the platform, accessing the platform (login issues), slow to load, inaccurate information)
    2. Are there intuitional barriers that may prevent you from using DHDR?
    3. What resources do you use to develop a BPMH
15. Have you ever used the drug profile viewer?
    1. How does the process differ for accessing DPV?
