# Supplementary material for: Understanding Engagement and the Potential Impact of an Electronic Drug Repository: Multi-Methods Study
Source: JMIR Form Res. 2022 Mar 30;6(3):e27158. doi: 10.2196/27158 (PMC9008523; doi:10.2196/27158)
Supplement: Multimedia Appendix 4 [file formative_v6i3e27158_app4.docx]

# **Appendix 4. Survey validation results for key experience domains.**

As our survey has not been validated, we explored preliminary convergent validity to test whether the key experience domains (i.e., usefulness, quality, training, and satisfaction) were measuring the same construct [29, 30]. The Shapiro-Wilk test was used to assess normality and Spearman’s Rho tests were applied to examine correlations within the domains [31]. *A priori*, we decided that negative statements within each domain were negatively correlated with positive statements which would be indicative of convergent validity.

| **DHDR User Experience Domains** | **Spearman Rho (r_s_) value** | **P-Value** |
| --- | --- | --- |
| Usefulness | -0.18 | 0.27 |
| Quality of data | -0.12 | 0.47 |
| Training | -0.30 | 0.07 |
| Overall satisfaction | -0.18 | 0.26 |

Results from the Shapiro-Wilk test suggested that non-parametric tests were appropriate as such Spearman's rho was conducted. Across all key domains, coefficients were negatively correlated, suggesting the survey items within each section were slightly convergent; however, the results were not statistically significant.
