# Supplementary material for: Understanding Engagement and the Potential Impact of an Electronic Drug Repository: Multi-Methods Study
Source: JMIR Form Res. 2022 Mar 30;6(3):e27158. doi: 10.2196/27158 (PMC9008523; doi:10.2196/27158)
Supplement: Multimedia Appendix 5 [file formative_v6i3e27158_app5.docx]

# **Appendix 5. Comparison of demographic information on survey for DHDR users and non-users (N=161).**

| **Demographic attribute** | **Number of DHDR users (%)** | **Number of DHDR non-users (%)** |
| --- | --- | --- |
| **Gender** |  |  |
| Male | 11 (27.5) | 19 (15.0) |
| Female | 29 (72.5) | 108 (85.0) |
| **Age category** |  |  |
| 18-34 | 10 (25.0) | 43 (34.0) |
| 35-49 | 12 (30.0) | 33 (26.0) |
| 50-64 | 17 (42.5) | 50 (39.3) |
| 65+ | 0 (0.0) | 1 (0.7) |
| Not reported | 1 (2.5) | 0 (0.0) |
| **Healthcare Setting** |  |  |
| Acute care | 25 (62.5) | 72 (56.7) |
| Primary care | 8 (20.0) | 16 (12.6) |
| Community care | 6 (15.0) | 29 (22.8) |
| Long-term care | 0 (0.0) | 2 (1.6) |
| Other | 1 (2.5) | 8 (6.3) |
| **Primary occupation** |  |  |
| Physician | 12 (30.0) | 13 (10.2) |
| Nurse | 6 (15.0) | 46 (36.2) |
| Pharmacist | 16 (40.0) | 29 (22.8) |
| Allied health professional | 0 (0.0) | 9 (7.0) |
| Support personnel | 2 (5.0) | 5 (3.9) |
| Administrative staff | 2 (5.0) | 4 (3.1) |
| Other | 2 (5.0) | 21 (16.5) |
| **Primary source of clinical information** |  |  |
| Hospital information system | 19 (47.5) | 52 (41.0) |
| Electronic Medical Record | 13 (32.5) | 28 (22.0) |
| Client Health and Related Information System | 1 (2.5) | 8 (6.3) |
| Paper records | 0 (0.0) | 22 (17.3) |
| Other | 4 (10.0) | 17 (13.4) |
| Not reported | 3 (7.5) | 0 (0.0) |
| **Provincial viewer** |  |  |
| ClinicalConnect | 17 (42.5) | 71 (56.0) |
| ConnectingOntario | 18 (45.0) | 7 (5.5) |
| None of the above | 5 (12.5) | 48 (37.8) |
| Not reported | 0 (0.0) | 1 (0.7) |
