# Supplementary material for: Understanding Engagement and the Potential Impact of an Electronic Drug Repository: Multi-Methods Study
Source: JMIR Form Res. 2022 Mar 30;6(3):e27158. doi: 10.2196/27158 (PMC9008523; doi:10.2196/27158)
Supplement: Multimedia Appendix 6 [file formative_v6i3e27158_app6.docx]

# **Appendix 6. Frequency of DHDR use among users (N=40).**

|  | **Number of users (%)** |
| --- | --- |
| **Access time (n=40)** | |
| <3 months | 2 (5.0) |
| 3-6 months | 28 (70.0) |
| 6-12 months | 3 (7.5) |
| >12 months | 7 (17.5) |
| **Frequency of use per week (n=39)** | |
| 0-4 times | 14 (35.9) |
| 5-9 times | 9 (23.0) |
| 10-14 times | 4 (10.0) |
| 15-19 times | 2 (5.0) |
| >20 times | 10 (25.6) |
| Not reported | 1 (2.6) |
