# Supplementary material for: Understanding Engagement and the Potential Impact of an Electronic Drug Repository: Multi-Methods Study
Source: JMIR Form Res. 2022 Mar 30;6(3):e27158. doi: 10.2196/27158 (PMC9008523; doi:10.2196/27158)
Supplement: Multimedia Appendix 7 [file formative_v6i3e27158_app7.docx]

# **Appendix 7. Mean scores and standard deviations on DHDR experience domains among survey respondents.**

| **Domain Question** | **Overall mean (SD)** | **Male mean (SD)** | **Female mean (SD)** | **P value** |
| --- | --- | --- | --- | --- |
| **Usefulness** | | | | |
| Constructing my patient's medication history using the information provided in the DHDR is difficult* | 4.18 (1.63) | 4.09 (1.51) | 4.21 (1.70) | 0.700 |
| Using the DHDR saves time when developing a best possible medication history | 4.98 (1.66) | 5.00 (1.90) | 4.97 (1.59) | 0.837 |
| The DHDR contains the features / information that I need to conduct a best possible medication history | 4.68 (1.65) | 4.55 (1.81) | 4.72 (1.62) | 0.792 |
| The DHDR fits well within my clinical routine/workflow | 4.25 (1.90) | 2.73 (1.85) | 4.83 (1.61) | **0.003** |
| **Quality of data contained in DHDR** | | | | |
| The DHDR does not contain the right data to establish a patient's medication history* | 4.13 (1.66) | 3.89 (1.69) | 4.21 (1.68) | 0.367 |
| The DHDR helps identify potentially harmful drug interactions / reactions | 3.63 (1.85) | 3.00 (1.61) | 3.86 (1.90) | 0.939 |
| The DHDR provides timely information on a patient's dispensed medication history | 4.73 (1.65) | 5.09 (1.58) | 4.59 (1.68) | 0.374 |
| The DHDR shortens the medical and / or nursing assessment time | 3.97 (1.68) | 3.45 (1.75) | 4.18 (1.63) | 0.396 |
| **Training and implementation of the DHDR** | | | | |
| The process to obtain access to the DHDR was simple and straightforward | 3.97 (1.84) | 3.09 (2.12) | 4.33 (1.62) | 0.694 |
| I obtained access to DHDR in a timely manner | 3.95 (1.89) | 2.82 (2.14) | 4.41 (1.60) | 0.646 |
| The training and materials I received on the DHDR was sufficient and easy to understand | 3.90 (1.65) | 3.36 (1.75) | 4.11 (1.59) | 0.445 |
| I often encounter technical issues when accessing the DHDR* | 4.72 (1.52) | 5.09 (1.51) | 4.57 (1.53) | 0.666 |
| **Satisfaction with the DHDR** | | | | |
| Access to the DHDR allows me to improve the quality of care I provide | 4.93 (1.51) | 5.45 (1.69) | 4.72 (1.41) | 0.790 |
| The DHDR has enhanced my ability to coordinate continued care | 4.60 (1.46) | 4.64 (1.69) | 4.59 (1.40) | 0.447 |
| The DHDR is confusing to use and navigate* | 4.77 (1.78) | 4.64 (1.96) | 4.82 (1.74) | 0.870 |
| Overall, I am satisfied with the DHDR | 4.33 (1.44) | 3.64 (1.75) | 4.61 (1.23) | 0.393 |
