# Supplementary material for: Understanding Engagement and the Potential Impact of an Electronic Drug Repository: Multi-Methods Study
Source: JMIR Form Res. 2022 Mar 30;6(3):e27158. doi: 10.2196/27158 (PMC9008523; doi:10.2196/27158)
Supplement: Multimedia Appendix 8 [file formative_v6i3e27158_app8.docx]

# **Appendix 8. Mean scores and standard deviations of the usefulness of current DHDR data Elements among DHDR users on survey (N=40).**

**Note**: Bars represent mean scores, and error bars represent the standard deviations.

**Abbreviations**: DIN drug identification number
