# Supplementary material for: Understanding Engagement and the Potential Impact of an Electronic Drug Repository: Multi-Methods Study
Source: JMIR Form Res. 2022 Mar 30;6(3):e27158. doi: 10.2196/27158 (PMC9008523; doi:10.2196/27158)
Supplement: Multimedia Appendix 9 [file formative_v6i3e27158_app9.docx]

# **Appendix 9. Rankings for new DHDR data elements among survey respondents.**

| **Data Element** | **Ranked 1st** | **Ranked 2nd** | **Ranked 3rd** | **Ranked 4th** |
| --- | --- | --- | --- | --- |
| Prescribed medications (publicly paid medications) | **19** | 12 | 6 | 0 |
| additional clinically relevant data elements | 2 | 10 | **18** | 8 |
| privately paid medications | 10 | **16** | 5 | 6 |
| Increasing deployment of the existing DHDR (in the absence of enhancements above) to a broader user group | **7** | 2 | 8 | **21** |

**Note:** Bold items represent the number of times participant rated an item and was used to determine rankings.
