# Supplementary material for: Understanding Engagement and the Potential Impact of an Electronic Drug Repository: Multi-Methods Study
Source: JMIR Form Res. 2022 Mar 30;6(3):e27158. doi: 10.2196/27158 (PMC9008523; doi:10.2196/27158)
Supplement: Multimedia Appendix 10 [file formative_v6i3e27158_app10.docx]

# **Appendix 10. Overall perceptions of the value and impact of the DHDR among female and male DHDR users (N=40).**

| **Survey Items** | **Overall**  **Mean (SD)** | **Male**  **Mean (SD)** | **Female**  **Mean (SD)** | **P-value** |
| --- | --- | --- | --- | --- |
| **Value of the DHDR** | | | | |
| How valuable is it to have access to prescribed medications when developing a best possible medication history? | 6.33 (0.89) | 6.73 (0.47) | 6.17 (0.97) | 0.3639 |
| How valuable is it to have access to dispensed medications when developing a best possible medication history? | 6.35 (0.80) | 6.64 (0.50) | 6.24 (0.87) | 0.547 |
| How valuable is it to have access to additional clinically relevant data elements when developing a best possible medication history? | 6.18 (1.01) | 6.45 (0.82) | 6.07 (1.07) | 0.4085 |
| How valuable is it to access privately funded medications when developing a best possible medication history? | 6.18 (1.03) | 6.18 (1.25) | 6.17 (0.97) | 0.6795 |
| How valuable is it to have DHDR integration into your hospital information system or electronic medical record developing a best possible medication history? | 6.25 (1.03) | 6.64 (0.92) | 6.10 (1.05) | 0.3515 |
| How valuable is it to have DHDR used more broadly to facilitate communication with healthcare providers? | 5.90 (1.22) | 6.00 (1.26) | 5.86 (1.22) | 0.726 |
| **Perceptions of the impact of the DHDR** | | | | |
| Do you feel that having access to DHDR reduced the need to reach out to other healthcare providers when developing a best possible medication history? | 4.21 (1.83) | 4.73 (1.85) | 4.00 (1.82) | 0.5375 |
| Do you feel that having access to DHDR reduces adverse drug events or inappropriate prescribing? | 4.29 (1.64) | 4.45 (1.97) | 4.22 (1.53) | 0.8063 |
| Do you feel that having access to DHDR improves patient outcomes? | 4.76 (1.36) | 5.18 (1.60) | 4.59 (1.25) | 0.8278 |
