# Supplementary material for: Understanding Engagement and the Potential Impact of an Electronic Drug Repository: Multi-Methods Study
Source: JMIR Form Res. 2022 Mar 30;6(3):e27158. doi: 10.2196/27158 (PMC9008523; doi:10.2196/27158)
Supplement: Multimedia Appendix 11 [file formative_v6i3e27158_app11.docx]

**Appendix 11. Outstanding quotes from qualitative interviews**

| **Key Finding** | **Quote** |
| --- | --- |
| **Clinical Use Cases** | |
| Clinical use case: Overdose | “People will come in and say that they took a whole bunch of medications, like they overdosed. And it’s really helpful if you know, at the very least, the dose of what they take…It really makes a difference as far as toxicity levels and that can change your plan of care.” – P30 |
| Clinical use case: Out of town patients | “I had a scenario where a woman came to town, she was visiting family, she forgot, she couldn’t remember the name of her atrial fib medication, she forgot it at home. She was only going to be there for a couple of days, her pharmacy was closed. Just no ability to help this poor woman who was experiencing an a-fib pretty much at the time she was in the store. Being able to look up on the DHDR what she was taking and provide an emergency supply of that medication was … that, to me, was one of the most valuable … we literally prevented a stay in the hospital, doing that.” – P29 |
| Clinical use case: Emergency | “It eliminates some of those patients who aren’t able to tell me what they’re on, aren’t able to tell me what pharmacy they go to, haven’t brought in any medications. It’s by far, for those situations, it’s unbelievably monumental. I honestly, working in Emerg on Sundays, I don’t know how I would get this information any other way. They would go without therapies for multiple days, more than likely. I think in those specific situations, I wouldn’t be able to do a BPMH without it.” – P11 |
| Value of NMS Data | “Someone came and asking for opioids for dental pain and didn’t really mention that she had been on opioids before. And so I looked in the medication list and she’d actually been prescribed opioids from numerous different Emergency Departments, and so I called the pharmacy just to verify that she had all these medications and I didn’t prescribe any more…” – P16 |
| **Perceived Impact & Awareness of DHDR** | |
| Educating providers about the DHDR to increase adoption | “Training all the outpatient prescribers to do medication reconciliation and BPMHs, I have to go one on one to them, to give them training in person, and that’s been the only way that they will engage in training. They don’t want to do another module, they don’t want to do all this…I think maybe sometimes even giving them access before they know exactly how to use it may be valuable, so that they could see the value, and then they’d do the training.” – P11 |
| Organizational barriers to adoption for some community pharmacists | “…the barrier to pharmacists accessing more information and doing more good things for people, means they do more stuff, more work. Which means that…it doesn’t fit the business models people who want pharmacists doing less stuff and less work, and just churning products…that’s the business that they’re in, and that’s the challenge of having the business part conflicting with what pharmacists can do and should do…But if you give somebody more tools to use, they’re going to do a better job…it’s going to take more time, and effort, and energy, and all this stuff, which is resources, which is money, which is creating a conflict.” – P8 |
| Suggestion of having different onboarding procedures depending on the organization | “I think a standard protocol connection should be written. There is one for hospitals that works. There isn’t one for pharmacy that would address the different ownership models. I think it would be, based on our experience, we could assist very quickly in writing that protocol to enable any pharmacy to connect. As it is today I think that protocol, whoever is at cSWO at the time interprets privacy differently and has different requirements. It’s a very fluid discussion that we’ve been having so I think nailing down that process for example with cSWO and then transporting it to the other two viewers, you could very quickly get community pharmacy connected across the board.” – P21  “It’s very simple and then you have kind of a toolkit that says if you’re independent or an associate owner this is the path you follow, and if you’re a corporate owner this is the path you follow then it’s simple but it’s just been nailing that process that has been frustrating” – P21 |
| Improved communication is required between providers looking to gain access and eHealth Ontario | “You basically go on the eHealth Ontario website and then you look at the link that talks about getting access for your clinic. Then you contact somebody and they usually will take their time to get back to you, but within maybe a month they’ll get back to you and they’ll put you on the waiting list and you’ll wait another month and then they’ll call you back to arrange a time. Kind of works like that. Then, they’ll come to your clinic to give you a little bit of orientation and then they’ll set it up.”- P5  “Yeah, I mean, just don’t make it something I have to adopt, just put it in my workflow. And then, if I’m knocking on your door trying to adopt it, like answer me and don’t make it so difficult.” – P24 |
| The case for community pharmacy access | “If the Ontario College of Pharmacists wants pharmacists to practice to their full scope in community, then it needs to be available in community.” – P25 |
| **Impact on Workflow and User Experience** | |
| The need for the sig/dosing regimen | “It’s particularly relevant for things like diuretics where like you may be dispensing a Lasix 20 milligram tablet, but the standard thing you’re telling the patient is ‘I want you to take 2 of these twice a day.’ So me as an Emergency Physician, I just see that they’re on Lasix 20 and if I’m going to renew their prescription or they’re saying what should I do tomorrow, I don’t know. If I don’t have what their active bottle is, actually I don’t if they’re taking 20 or 40 and I make the wrong assumptions and I refill their prescription for 20 once a day and really they’re taking 40 twice a day and that leads to real harm. We make big mistakes both ways about that so that’s very frustrating.” - P12 |
| Views on lack of EMR integration | “As you can imagine, somebody…is doing a consult, they’re opening up their computer, they’re looking through [their EMR]…But then, in order to get to ConnectingOntario you have to actually open up a different window…you actually have to come out of [the EMR] to load up another window, which takes you away from what you were doing before…That takes time. It takes time to load. So, I think you should…actually integrate it into an EMR system, so that the information can be accessed easily instead of through the ConnectingOntario interface.” – P5 |
| Views on the lack of completeness of drug data | “Some of the newer medications that are on the market are not covered by the ministry, and so… it gets missed. I’ve seen BPMHs created by nurses, that have missed those drugs, and then after, where digging through … speaking to the community pharmacy, they come up, something that was dispensed. That has led to a delay in therapy being continued” – P27  “Obviously it’s a gap but hopefully this could be like a piece and a step towards getting a more complete record at some point. Even knowing that it has a gap it’s still useful. It’s still less of a gap than I have without it.” – P24 |
| Impact of non-centralized data on provider workflow | “I think it adds to the workflow, it stops and you may have to go back to the patient or you have to go to these other resources, a community pharmacist or you have to go to the family doctor to get that information. Whereas if I had access directly I'm saving everybody else time too.” – P23  “It would be nice if everything was included in there. That would be great if it could be added. But we are so used to asking that it doesn't become a really big problem.” – P23 |
